# Musculoskeletal ultrasound in the emergency department: a narrative review for general radiologists

**DOI:** 10.1007/s10140-025-02422-6

**Published:** 2025-12-16

**Authors:** Federico Pistoia, Marta Macciò, Riccardo Picasso, Federico Zaottini, Maria Elena Susi, Giovanni Marcenaro, Carlo Martinoli

**Affiliations:** 1 IRCCS Ospedale Policlinico San Martino, Largo Rosanna Benzi, 10, Genoa, Italy; 2 Department of Health Sciences (DISSAL), Radiology Section, University of Genova, Via Pastore 1, Genoa, Italy

**Keywords:** Musculoskeletal ultrasound, Emergency department, Trauma, Diagnostic imaging

## Abstract

Ultrasound (US) is a non-invasive, radiation-free imaging modality ideal for evaluating several acute musculoskeletal conditions in the emergency department (ED). This narrative review provides general radiologists with a concise guide to high-yield MSK US applications in the ED, including tendon ruptures, muscle injuries, bursitis, soft tissue infections, hematomas, and occult fractures. It highlights clinical presentations, sonographic findings, and technical tips to enhance diagnostic confidence and support timely management.

## Introduction

Musculoskeletal (MSK) disorders are a frequent reason for presentation to the emergency department (ED), encompassing a wide spectrum of conditions ranging from minor soft tissue injuries to limb-threatening infections or tendon ruptures. While radiography remains the initial imaging modality for many MSK issues, it lacks sensitivity and specificity for soft tissue abnormalities. In this context, high frequency US has emerged as an invaluable imaging tool, offering high-resolution evaluation of tendons, muscles, and subcutaneous tissues. The advantages of US in the ED setting are well established: it is non-invasive, radiation-free, and widely available. Despite these benefits, many emergency and general radiologists may feel less confident in performing and interpreting MSK US due to the specific skills it requires. This is particularly relevant in emergency contexts where tempestive diagnosis can significantly influence management and outcomes. This review aims to provide a practical and concise guide to the most relevant, high-yield applications of MSK US in the ED. It is primarily intended for general radiologists, while remaining relevant to other clinicians involved in acute musculoskeletal care, whether conducted within the radiology department or at the bedside. The applications of US covered in the article include the assessment of tendon ruptures, muscle injuries, bursitis, soft tissue infections, and hematomas, all of which are frequently seen in emergency settings. Emphasis is placed on typical clinical presentations, characteristic sonographic findings, and technical tips to improve diagnostic confidence.

### Overview of common musculoskeletal ultrasound techniques

An effective MSK US examination relies on appropriate probe selection, scanning orientations, and dynamic maneuvers to optimize diagnostic accuracy and procedural safety. High-frequency linear transducers (typically 7–15 MHz or higher) are standard for superficial structures, while lower-frequency curvilinear transducers may be used for deeper tissues or in obese patients. Scanning orientations include longitudinal and transverse views, which are employed differently depending on the structure and pathology examined, often with comparison to the contralateral side to distinguish normal from abnormal findings. Dynamic examination is a hallmark of MSK US, allowing real-time assessment of joint movement and improving evaluation of various pathologies, particularly tendon integrity. Different dynamic techniques exist for major joints, often involving patient movement or stress maneuvers to reveal structures (e.g. supraspinatus tendon in the shoulder) or pathology not readily visible on static images. Additional techniques, such as graded compression and Doppler imaging, help differentiate soft tissue lesions and enhance vascular assessment (Table 1).

**Table 1:** Ultrasound assessment and management of most common tendon lesions and acute muscle tears

Lesion	Typical Location	How to Scan	Ultrasound Appearance	Clinical Notes	When to Escalate
Achilles Tendon Rupture	Proximal, mid-substance, or distal insertion	Patient prone with feet hanging; scan from calcaneus to musculotendinous junction; dynamic plantar-/dorsiflexion	Complete fiber discontinuity with hypoechoic gap and retraction; absent motion on dynamic test	Common injury, especially in athletes; sudden onest of pain and weakness in the posterior lower leg	MRI if: diagnostic uncertainty, complex/chronic injury, or pre-op planning needed (degeneration, rupture site/shape, retraction). Influences minimally invasive vs. open repair
Distal Biceps Tendon Rupture	At radial tuberosity insertion	Elbow flexed 90°, forearm supinated; anterior/medial approach	Hypoechoic defect with proximal tendon retraction and surrounding hematoma	Sudden pain and weakness in elbow flexion and forearm supination; “Popey” deformity	MRI to better define partial vs. complete tear; orthopedic consult for surgical repair
Quadriceps Tendon Rupture	Suprapatellar region near superior patellar pole	Supine, knee slightly flexed; long- and short-axis	Partial: focal hypoechoic defect; Complete: disruption of all layers with hematoma	Often in older or diabetic patients; associated with sudden extension failure	MRI if: diagnostic uncertainty, discrepancy between clinical findings and US, or when more detailed assessment of the extent, location, and complexity of the tear is needed to guide surgical planning
Patellar Tendon Rupture	Usually at inferior patellar pole	Supine, knee flexed ≈ 30°; scan from patella to tibial tuberosity	Hypoechoic gap or discontinuity; possible bony avulsion	Younger active patients; loss of active extension	MRI/CT for avulsion or to better differentiate between complete and partial lesion; orthopedic consult for high-grade or complete tears
Rotator Cuff Tear (acute)	Most often supraspinatus; less commonly subscapularis or infraspinatus	“Modified Crass” or external-rotation views; assess in both planes	Full-thickness: fluid-filled gap; Partial: focal fiber disruption with loss of fibrillar pattern	After trauma or degenerative; supraspinatus tear correlates with impaired arm abduction and elevation	MRI for complete/multi-tendon tears; orthopedic referral for repair assessment
Finger Flexor Tendon Rupture	Flexor digitorum profundus/superficialis or flexor pollicis longus	Volar longitudinal and transverse scans; dynamic finger motion	Loss of tendon continuity and retraction; absent gliding	Usually traumatic; urgent repair improves outcome	Immediate hand-surgery consult for complete rupture; MRI if stump not seen
Pectoralis Major Tendon Rupture	Myotendinous junction or humeral insertion	Arm abducted and externally rotated; scan over bicipital groove and move medially	Focal or complete fiber discontinuity with hematoma and retraction	Common in weightlifters; deformity of anterior axillary fold	MRI to determine the exact extent and anatomical location of the lesion (tendinous vs. myotendinous junction); orthopedic referral for repair assessment
Triceps Tendon Rupture	Myotendinous junction or olecranon insertion	Posterior elbow approach, flexed 90°; long-axis	Disrupted fibrillar pattern with fluid or hematoma; possible bony avulsion	Rare; often post-traumatic or steroid-related	MRI for uncertain extent; orthopedic referral for repair assessment
Acute Muscle Tear	Myotendinous junctions of major muscle groups (e.g. calf, hamstring)	Long- and short-axis over area of pain;	Hypoechoic or heterogeneous area of fiber disruption ± hematoma or fluid tracking along fascial planes	Graded by fiber disruption (mild to complete); clinically may mimic DVT or contusion	MRI for deep or in low grade lesion (better sensibility than ultrasound); rehab/orthopedic consult for high-grade or recurrent injuries

### Muscle and tendon injuries

#### Achilles tendon rupture

**Fig. 1:**
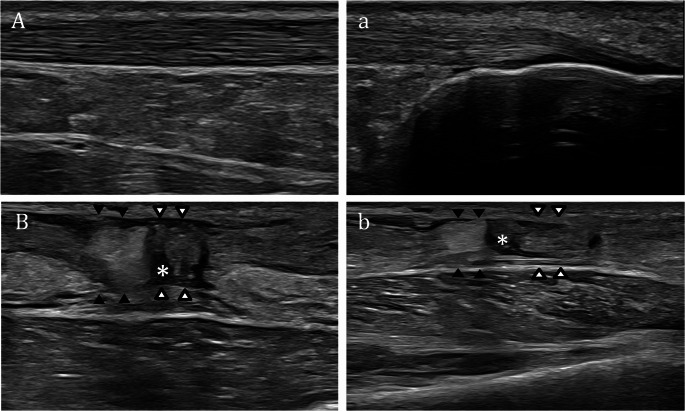
Complete rupture of the Achilles tendon at the mid-portion. **A–a **Sagittal US images show a normal fibrillar Achilles tendon at its mid-portion (A) and calcaneal insertion (a). **B–b **Long-axis images obtained during plantar flexion (B) and dorsal flexion (b) of the foot to magnify the separation of the torn ends of the Achilles tendon. A gap between the proximal (black arrowheads) and distal (white arrowheads) stumps is filled with anechoic fluid (asterisks), consistent with hematoma in the tendon bed

Achilles tendon rupture is a common injury, especially in athletes, and requires accurate diagnosis for optimal recovery. US is highly sensitive for detecting acute ruptures, particularly when physical examination is limited by pain or swelling, demonstrating tendon discontinuity, retracted ends, hematoma, and loss of the normal fibrillar pattern [1–3]. Achilles tendon ruptures are typically categorized based on their anatomical location into three main types: (i) proximal ruptures at the musculotendinous junction, (ii) mid-substance tears involving the free tendon, and (iii) distal ruptures at the calcaneal insertion (enthesis). Among these, the most frequently reported site of rupture is the mid-tendon region, located approximately 4–6 cm proximal to its insertion on the calcaneus [4]. Sonographic assessment of the Achilles tendon is typically performed with the patient in the prone position, feet hanging over the edge of the examination table. This position facilitates both passive and active plantarflexion and dorsiflexion of the foot, which is essential for dynamic evaluation. Long-axis imaging is used to assess the tendon and surrounding structures during these movements, aiding in the detection of abnormalities in tendon continuity. The plantaris tendon, located medial to the Achilles, may be visualized in short axis as a small oval structure. US evaluation in the acute phase can be technically challenging due to pain, swelling, and hematoma formation, which may obscure the site of tendon discontinuity. In particular, hematoma or edema between torn tendon stumps may appear isoechoic and fill the defect, making it difficult to detect the rupture. A systematic long-axis scan extending from the distal enthesis at the calcaneus to the proximal musculotendinous junction of the soleus and gastrocnemius muscles is essential to localize the tear and assess its extent. Dynamic maneuvers can enhance diagnostic accuracy: during dorsiflexion from a plantarflexed position, the lack of synchronous motion between tendon stumps suggests a complete rupture (Fig. 1.) Observing tendon edge displacement with movement increases diagnostic confidence, especially when static imaging findings are equivocal [5].

#### Distal biceps tendon rupture

**Fig. 2:**
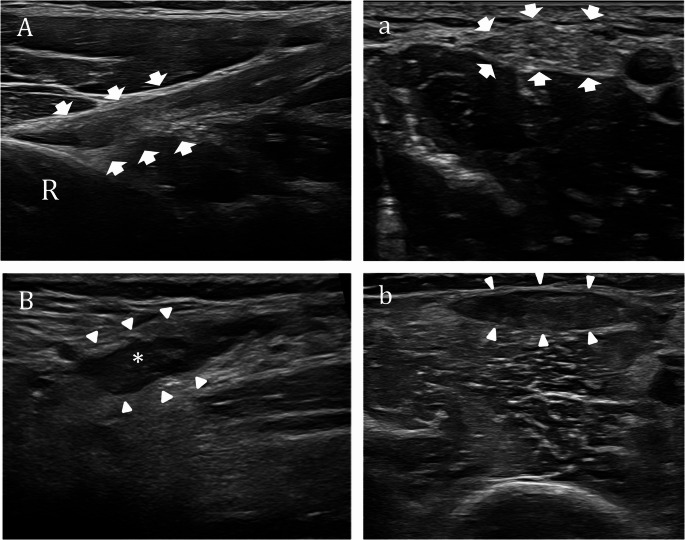
Complete tear of biceps tendon. **A-a** Long-axis and short-axis US images show a normal distal biceps tendon (white arrows) inserting on the radial tuberosity (R). **B-b** Long-axis and short-axial US images demonstrate a complete tear of the distal biceps tendon (arrowhead). B The long –axis US image show hypoechoic fluid (asterisks) filling the distal bed of the retracted biceps tendon; the tendon itself is not clearly visualized. b Short-axis US image demonstrate the torn and retracted tendon at distal portion of the arm. *R: radius*

Distal biceps tendon rupture typically affects middle-aged individuals, often during eccentric biceps contraction, and is strongly associated with male sex, smoking, and elevated BMI. Clinically, it presents with sudden pain, weakness in forearm supination, visible biceps deformity, and bruising [6]. US is an accurate tool for diagnosing distal biceps tendon ruptures, especially when combined with thorough clinical assessment and multiple scanning approaches. Four main US approaches are used—anterior, medial, lateral, and posterior—with operator preference influencing choice. The medial approach is often favored, but the anterior approach with the forearm pronated is also common. For less experienced operators, the medial approach is generally recommended. With the elbow flexed at 90° and the forearm supinated, the probe is placed longitudinally and medially over the distal arm. After identifying the brachial artery, the probe is shifted laterally to visualize the biceps tendon in its long axis. In case of tendon rupture, US typically reveals a loss of the normal fibrillar pattern, with a visible gap or discontinuity at the distal biceps tendon insertion site, often replaced by a hypoechoic area representing hematoma or fluid collection [7, 8]. The ruptured tendon may appear retracted proximally, sometimes with a visible mass or “balling up” of the muscle belly. Hypoechoic or anechoic regions adjacent to the rupture site indicate hematoma or fluid effusion (Fig. 2). Flexion and extension along with pronosupination maneuvre help differentiate between partial and complete tendon ruptures.

#### Quadriceps tendon rupture

**Fig. 3:**
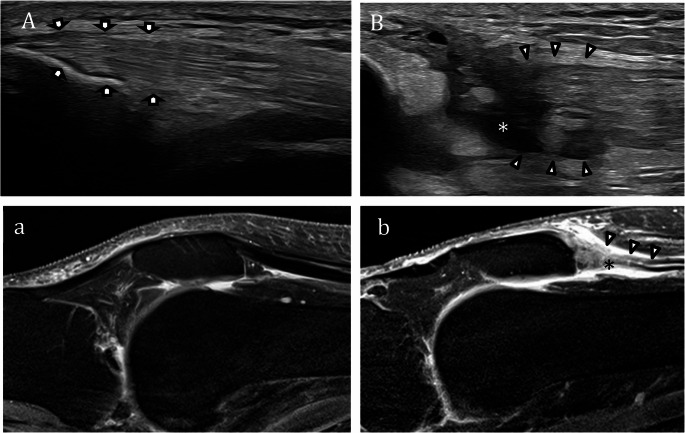
Complete tear of the quadriceps tendon** A-a** Longitudinal US and sagittal MRI PD fat-sat images demonstrate normal appearance of the quadriceps tendon (arrows) at its insertion onto the patella. **B-b** Longitudinal US image and sagittal fat saturation PD MRI image demonstrate a complete tear of the quadriceps tendon, with proximal retraction of the tendon stump (arrowheads). A surrounding hypoechoic (US) and hyperintense (MRI) fluid collection (*) is visible

Quadriceps tendon rupture is an uncommon injury, typically affecting men over 40 and often linked to conditions such as diabetes, gout, or chronic steroid use [9]. It usually presents with sudden knee pain, inability to extend the knee, and a palpable gap above the patella. While diagnosis is primarily clinical, imaging—particularly US and MRI—can accurately assess the extent of the injury. Partial tears may be treated conservatively, but complete ruptures require prompt surgical repair to restore function and prevent long-term disability. US represents a valuable initial modality for diagnosing quadriceps tendon rupture, especially when clinical assessment is inconclusive due to pain or swelling. US can accurately identify both complete and partial tears by visualizing disruption of the tendon fibers and associated hematoma, with high sensitivity but somewhat lower specificity compared to MRI, which remains the gold standard for definitive diagnosis and for resolving equivocal cases or suspected normal anatomical variants [10, 11]. US is particularly useful in the ED, allowing for immediate assessment and differentiation between partial and complete ruptures, which is crucial for timely surgical intervention and optimal outcomes [12].The superficial location of the quadriceps tendon makes it well-suited for sonographic evaluation, and dynamic imaging can further aid in assessing tendon function. The quadriceps tendon is a multilayered structure, with most studies identifying three or four distinct layers. The superficial layer is formed by the rectus femoris tendon, the middle layer(s) by the vastus medialis and vastus lateralis, and the deepest layer by the vastus intermedius tendon. Some studies have described a consistent three-layered arrangement, while others have found four layers, especially when accessory muscle heads are present, and even more complex variations in rare cases [13–16]. On US, a normal quadriceps tendon appears as a thick, linear, and echogenic structure inserting onto the patella. Partial ruptures may involve one or more of the tendon layers and typically present as focal hypoechoic defects within the tendon, sometimes with preserved continuity in the unaffected layers. In contrast, complete ruptures show disruption of all tendon layers, with the tendon ends separated by a hypoechoic or anechoic hematoma (Fig. 3). US can help distinguish between partial and complete tears by identifying which layers are involved and the extent of fiber disruption, though operator experience and patient factors (such as obesity) can affect accuracy. However, as previously mentioned, MRI is needed in some cases for precisely identifying the specific layer involved and guiding management decisions, as the number and thickness of injured layers influence treatment strategies and prognosis [17].

#### Patellar tendon rupture

Rupture of the patellar tendon is an uncommon yet severe injury, predominantly seen in physically active individuals under 40 [18, 19]. It usually results from a sudden, forceful extension of the knee against resistance—such as during jumping or sprinting—and often affects tendons compromised by chronic overuse, systemic conditions, or corticosteroid exposure [20]. Typical clinical findings include acute pain, knee swelling, an inability to actively extend the leg, and a high-riding patella (patella alta) evident on imaging, along with a palpable defect just below the patella. Patellar tendon ruptures most commonly occur as avulsion injuries at the inferior pole of the patella, accounting for over 80% of cases, while midsubstance and tibial avulsion tears are much less frequent [21]. The standard scanning approach of the patellar tendon involves both longitudinal and transverse scans, typically with the patient in a supine or standing position and the knee slightly flexed to optimize visualization of the tendon from the patella to the tibial tuberosity. Sonographic findings of patellar tendon rupture typically include a hypoechoic area within the tendon, indicating a tear, often accompanied by thickening of the tendon. In cases of partial rupture, US can quantify the lesion by measuring the length of the hypoechoic defect, which helps in grading the severity: Grade I (< 10 mm), Grade II (10–20 mm), and Grade III (>20 mm) lesions, with larger lesions correlating with a higher likelihood of requiring surgical intervention [22]. A cone-shaped, poorly echogenic area exceeding 0.5 cm in length in the center of the patellar tendon, along with localized thickening, is a reliable indicator of partial rupture or “jumper’s knee” [23]. In complete ruptures, US demonstrates a distinct gap in the tendon with retracted tendon ends, often accompanied by surrounding soft tissue swelling or hematoma in the tendon bed. In acute traumatic cases, sonography can also reveal avulsed bony fragments, and associated hematoma, all of which correlate well with surgical findings. Notably, studies consistently demonstrated excellent intra- and inter-rater reliability for US assessment of patellar and quadriceps tendon, even among operators with varying experience [24–26].

#### Rotator cuff tendon ruptures

**Fig. 4:**
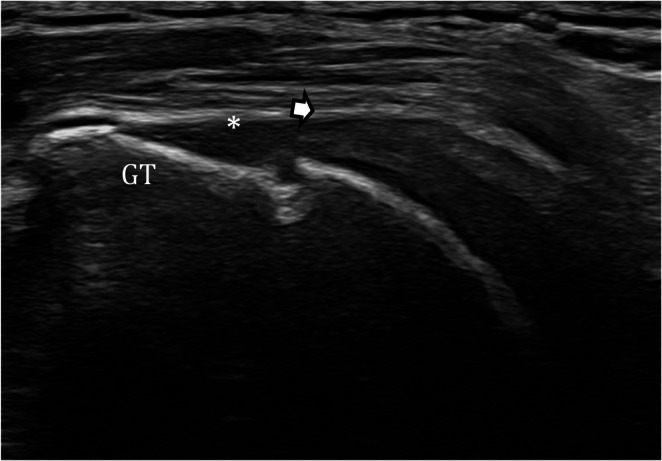
Full-thickness supraspinatus tear. Long-axis US image demonstrates a fluid-filled defect (asterisk) at the former insertion site of the supraspinatus tendon on the greater tuberosity (GT), with proximal tendon retraction (white arrows), consistent with a large full-thickness supraspinatus tear. *GT: greater tuberosity*

Acute rotator cuff rupture is a sudden tear of the shoulder’s rotator cuff tendons, most often following trauma. Early surgical repair, ideally within 3 weeks, is associated with better functional outcomes, particularly in younger patients and traumatic tears. For small acute tears, both surgical repair and physiotherapy can achieve comparable short-term results, although nonoperative management carries a risk of tear progression over time [27–29]. The supraspinatus tendon is most frequently affected in acute rotator cuff tears, often presenting as a full-thickness rupture following trauma such as a fall [30]. Acute tears more commonly involve the subscapularis than degenerative lesions, with reported involvement in over 60% of full-thickness acute tears [31]. The infraspinatus and teres minor are less frequently involved in isolation but may be affected in larger or multi-tendon injuries [30, 31]. High-resolution US shows excellent diagnostic performance for full-thickness supraspinatus tears, with reported sensitivities ranging from 94% to 97% and specificities from 92.5% to 100%, closely matching the diagnostic accuracy of MRI [32, 33]. US evaluation of the subscapularis has a high specificity (often above 88%) but variable and generally lower sensitivity, especially for smaller or partial-thickness tears [taso^34^]. Optimal US examination of the rotator cuff tendons requires proper patient positioning: the supraspinatus is best assessed with the arm internally rotated and extended in the “modified Crass” position, whereas the subscapularis is evaluated with external rotation [35]. Both longitudinal and transverse scans should be obtained. Full-thickness tears appear as complete tendon discontinuity with fluid in the gap, whereas partial-thickness tears present as focal hypoechoic defects or fiber disruption (Fig. 4). In complete ruptures, tendon retraction and muscle trophism should be assessed to help distinguish acute from chronic tears. Therefore, report should specify tear presence, depth, location, and extent, noting insertional involvement or retraction, as well as associated muscle atrophy or fatty infiltration when visible.

#### Finger flexor tendon ruptures

Finger flexor tendon ruptures most commonly involve the flexor digitorum profundus, flexor digitorum superficialis, and flexor pollicis longus tendons, and can result from various mechanisms such as crushing injuries, lacerations, hyperextension, or forced flexion against resistance [36]. The majority of ruptures occur at the tendon insertion or musculotendinous junction, but midsubstance ruptures have also been reported, sometimes without any predisposing disease or degeneration [36, 37]. Immediate re-repair is recommended for ruptured primary flexor tendon repairs in the fingers, particularly in zones 2 and 1 (respectively from the distal palmar crease to middle of the middle phalanx and distal to the middle of the middle phalanx), to maximize the chance of functional recovery and minimize complications from delayed intervention. Therefore, immediate imaging has a role for suspected finger tendon ruptures in the ED, especially when clinical assessment is inconclusive or injury severity is unclear. US has demonstrated high sensitivity and specificity for detecting complete tendon lacerations, and can also assess tendon gliding and retraction, which are important for surgical planning [38]. The US examination should be performed in both axial (transverse) and sagittal (longitudinal) planes: the probe is placed transversely and then longitudinally along the volar aspect of the finger; comparison with a normal finger can be helpful for non-expert operator [39]. Dynamic assessment is crucial: passive and active flexion and extension of the finger help visualize tendon movement and identify discontinuity, retraction, or lack of gliding, which are signs of rupture [40]. The distal tendon stump can be seen moving with passive joint motion, while the proximal stump is visualized during active flexion; gaps, loss of continuity, or abnormal tendon position confirm rupture.

#### Other tendon ruptures

Although rotator cuff and biceps tendon injuries represent the most frequently encountered tendon pathologies of the shoulder and upper arm, other tendon ruptures, though less common, must be recognized for their distinct clinical, anatomical, and imaging features [41]. Among these, pectoralis major and triceps brachii tendon ruptures are increasingly reported, particularly in active individuals engaged in high-demand physical activity such as weightlifting. Awareness of their typical mechanisms of injury, anatomical characteristics, sonographic appearances, and management options is important to ensure timely diagnosis and optimal functional outcomes.

#### Pectoralis major tendon rupture

**Fig. 5:**
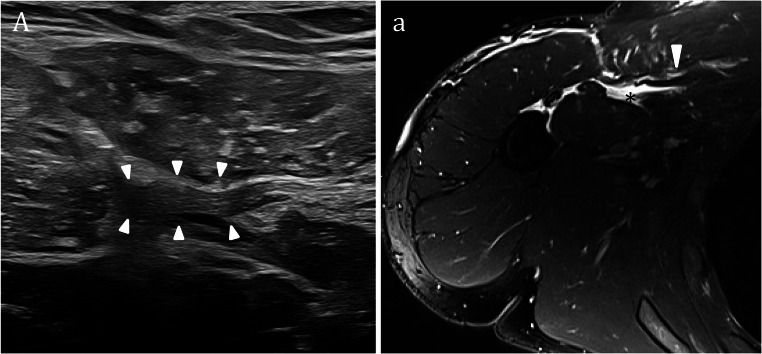
Complete tear of pectoralis major tendon.** A-a** Axial US image and axial fat saturation PD MRI images demonstrate a full-thickness tear of the pectoralis major tendon (arrowhead) with complete retraction from the humeral insertion and associated fluid collection (*)

Pectoralis major tendon rupture is an increasingly recognized injury that mainly affects young active men during eccentric muscle contractions, such as those in weightlifting. Common signs of pectoralis major tendon rupture include swelling, bruising, and deformity with loss of the anterior axillary fold. Patients often report weakness with pushing or arm adduction and may exhibit a dropped nipple or muscle bulge from retraction. Once swelling subsides, an indentation or asymmetry with medial retraction of the muscle belly becomes visible. The pectoralis major tendon inserts onto the lateral lip of the bicipital groove of the humerus, with a broad, flat footprint averaging about 73.3 mm in length and 3.3 mm in width [42]. The tendon results from the convergence of the two muscle heads: the clavicular and the sternal. The clavicular head is generally shorter and inserts more superiorly, whereas the sternal head is longer and inserts more inferiorly [43, 44]. Pectoralis major tendon ruptures most commonly occur at or between the myotendinous junction and the tendinous insertion on the humerus [45]. Optimal sonographic evaluation of the pectoralis major tendon is performed with the patient’s arm in slight abduction and external rotation. Scanning in both longitudinal and transverse planes allows for accurate identification of the distal tendon, which lies superficial to the long head of the biceps brachii tendon and inferior to the subscapularis. In the normal state, US demonstrates a fan-shaped convergence of muscle fibers twisting into a single tendon at its humeral insertion. The clavicular and sternal heads of the tendon can often be distinguished as separate echogenic components converging at the insertional footprint. Tendon tears results in disruptions of the normal fibrillar echotexture: as in the other tendon tears, partial-thickness tears typically appear as areas of reduced echogenicity with architectural disorganization, whereas full-thickness tears are characterized by complete fiber discontinuity, tendon retraction, and, in most cases, associated hematoma formation (Fig. 5). Operative repair is favored for most pectoralis major tendon and myotendinous junction injuries in active individuals, offering superior strength, function, and cosmesis compared to conservative management, though with some risk of complications. Nonoperative treatment may be considered for partial tears, muscle belly injuries, or low-demand patients, but often leads to persistent strength deficits and cosmetic concerns [46].

#### Triceps tendon rupture

**Fig. 6:**
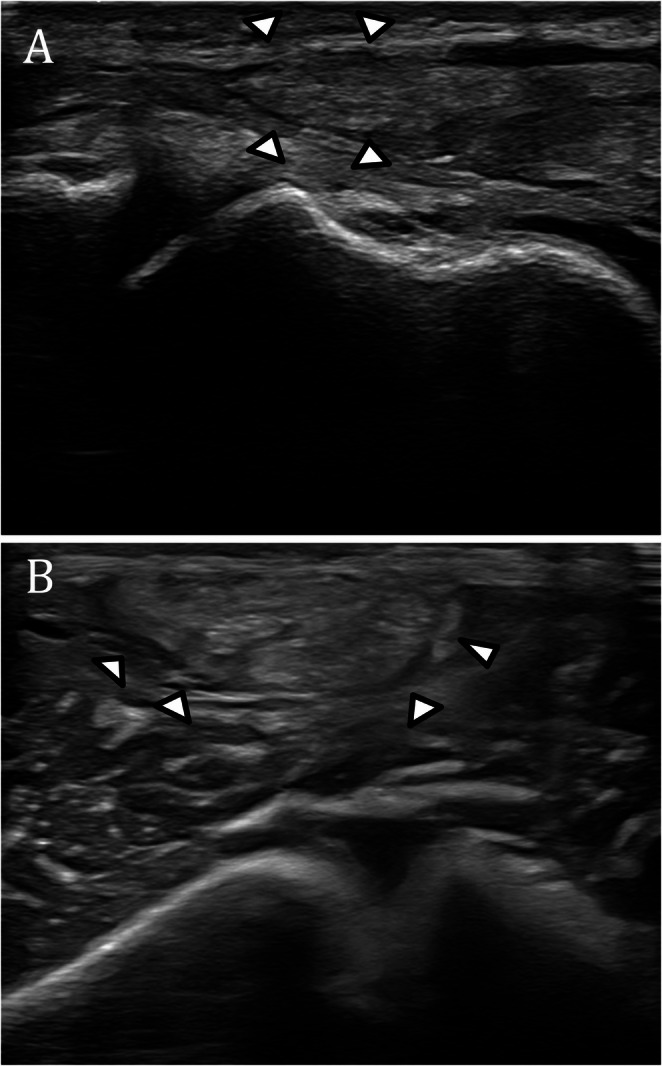
Partial tear of the medial head of the triceps tendon.** A-B** Long axis and short axis US images demonstrate a partial tear at the myotendinous junction of the medial head of the triceps tendon. Medial tendon stump appears thickened and with heterogeneous echotexture (white arrowheads)

Triceps tendon rupture is a rare injury (less than 1% of all tendon ruptures related to the upper extremity [47]), typically affecting middle-aged men, athletes, and weightlifters [48]. It usually results from a fall on an outstretched hand, forceful eccentric contraction, or direct elbow trauma [49]. As with other tendon ruptures, risk factors include anabolic steroid use, local corticosteroid injections, chronic renal failure, and metabolic bone disorders. Ruptures most commonly occur at the tendon’s insertion on the olecranon but can also involve the myotendinous junction [50]. Patients typically present with sudden posterior elbow pain, swelling, bruising, and weakness or inability to extend the elbow. A palpable gap above the olecranon and “boggy” swelling may be noted. The “flake sign” on lateral X-rays suggests bony avulsion. The triceps brachii tendon is formed by the convergence of its long, lateral, and medial heads, which have distinct origins. Anatomical studies have shown that the medial head has a separate, deeper insertion, while the long and lateral heads form a common superficial tendon [51]. All three components ultimately merge into a single unit at the bone. On US, the triceps tendon is best evaluated with the elbow flexed at 90°, both at rest and during dynamic maneuvers. Scanning is performed in longitudinal and transverse planes via a posterior approach, from the myotendinous junction to the olecranon insertion. The tendon is identified by its typical hyperechoic, fibrillar echotexture, and flexion-extension and medio-lateral movements aid in distinguishing the medial (deep) and lateral (superficial) tendon components. US reliably differentiates partial from complete tears and can detect selective involvement of individual heads, influencing clinical management [52]. Rupture is indicated by disruption of fibrillar architecture, tendon thickening, retraction, and hypoechoic or anechoic areas suggestive of hematoma. In avulsion injuries, bony fragments may be visualized (Fig. 6).

#### Acute muscle tear

Muscle tears are common sports injuries, often affecting muscles involved in eccentric contractions, such as the hamstrings and gastrocnemius [53]. They result from excessive stretching during muscle activation, typically causing tears at the myotendinous junction. Symptoms include acute pain and weakness, and diagnosis is usually based on clinical examination, with imaging reserved for unclear cases. On US, acute muscle tears may appear as hyperecogenic areas with distortion of normal architecture, indicating edema or hemorrhage, or as anechoic/hypoechoic areas of fibers disruption, which often represent more severe injuries with hematoma formation; compound lesions can show both features and suggest moderate to severe injury. Intrinsic muscle injuries, caused by simultaneous muscle contraction and elongation leading to myofiber damage, are classified into three grades based on US appearance [54, 55] (Table 2). Grade 1 injuries, characterized by minimal muscle tissue damage (< 5%), typically heal within 1–2 weeks. Grade 2 injuries involve partial lesions (>5%) without affecting the entire muscle. Grade 3 injuries, marked by complete rupture at the myotendinous junction associated with hematoma, require 5–8 weeks of recovery to reduce the risk of recurrence, the primary complication of these injuries [56].One of the most frequent muscle tear encountered in the ED is the strain or rupture of the medial head of the gastrocnemius muscle, often referred to as “tennis leg” [57, 58]. Sonographic findings in tennis leg typically reveal disruption of medial gastrocnemius aponeurosis or distal myoaponeurotic junction, with or without fluid collection between the medial gastrocnemius and soleus muscles, predominantly involving the myotendinous junction. The plantaris tendon, once thought to be a primary culprit of tennis leg, is now recognized as less frequently involved, with most cases attributable to the gastrocnemius [57–59]. In this context, US is frequently required to confirm the diagnosis of tennis leg, differentiate it from other causes of calf pain, such as deep vein thrombosis, which may sometimes be concomitant, and to grade the severity of the injury. Both longitudinal and short-axis ultrasound scans are recommended for the evaluation of muscle tears, particularly in the acute setting where sonographic findings may be subtle and most evident on one plane rather than the other.

**Table 2:** Ultrasound grading of intrinsic muscle injuries

Grade	Ultrasound findings	Prognosis
0	No evidence of muscle injury	Normal recovery
1	Small areas of damaged muscle tissue (< 5%)	Heals in 1–2 weeks
2	Partial lesion (> 5%) not involving entire muscle	Variable recovery; monitor for complications
3	Complete rupture at myotendinous junction	5–8 weeks recovery to reduce recurrence risk

#### Bursitis

**Fig. 7:**
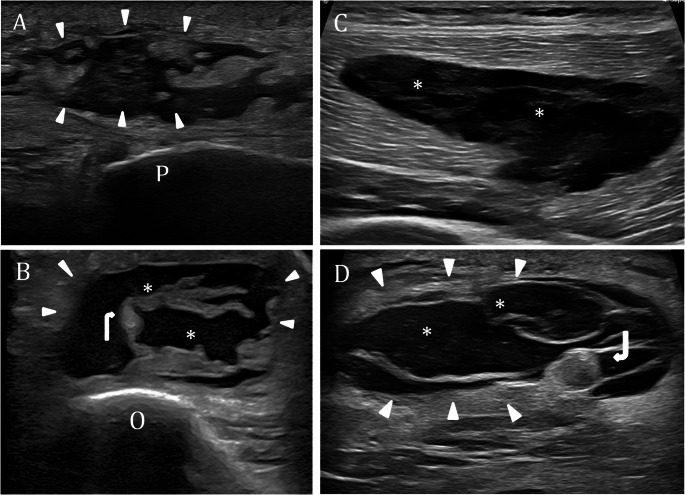
Soft tissues pathological findings. Prepatellar bursitis A short axis US image obtained over the patella (P) shows an anechoic subcutaneous effusion consistent with a distended prepatellar bursa (arrowheads). Olecranon bursitis B short-axis US image obtained over the olecranon process (O) demonstrates a distended olecranon bursa (arrowheads) containing thick septa (curved arrows) Intramuscular hematoma of the thigh C extended field-of-view axial US image of a young woman presenting with acute swelling and pain in the anterolateral thigh reveals a large, heterogeneously hypoechoic collection (asterisk) within the vastus lateralis muscle, consistent with an intramuscular hematoma. Morel-Lavallée lesion D short-axis US image demonstrates a subcutaneous fluid collection (asterisk) with an echogenic fat lobule (curved arrows), characteristic of a Morel-Lavallée lesion. *P: patella; O: olecranon process*

Bursitis is a common MSK complaint in the ED, usually presenting as acute swelling and localized pain over synovial bursae near major joints—most often the knee or olecranon bursa of the elbow [60]. It is often linked to repetitive motion, trauma, or local inflammation, and may present with swelling, tenderness, erythema, or warmth. US easily demonstrates bursal fluid collections, distinguishing bursitis from other causes of joint pain and swelling, such as joint effusions or cellulitis, and directly influencing patient management decisions, including guiding aspiration or injection procedures [61, 62]. On US, bursitis typically appears as a well-defined and compressible anechoic fluid collection within the expected location of a bursa. The bursal wall may appear thin and smooth; however, mild wall thickening, internal septations, or mural nodules can be observed in chronic or complicated cases of bursitis. The fluid content may range from simple (anechoic) to complex, containing echogenic debris. Doppler imaging may demonstrate increased vascularity along the bursal wall, suggestive of active inflammation [63]. Precise anatomical localization is essential for diagnosis, as in Gruberi bursitis, located between the extensor digitorum longus tendons and the dorsolateral aspect of the talus, or in ischial bursitis, where the fluid collection lies superficial to the ischial tuberosity. In the knee, anserine bursitis typically appears on US as a well-defined, anechoic fluid collection with smooth margins, located between the pes anserinus tendons and the medial collateral ligament. A particular form of complicated bursitis is the rupture of a Baker’s cyst, which often presents with acute calf pain and swelling, potentially mimicking more serious conditions such as deep vein thrombosis. US typically reveals a collapsed or irregular Baker’s cyst in the posteromedial aspect of the knee with hypoechoic fluid tracking along the intermuscular planes of the calf, most commonly between the gastrocnemius and soleus muscles. These fluid collections often communicate with a residual cystic structure at the back of the knee, confirming the diagnosis of cyst rupture. Differentiating bursitis from other soft tissue lesions relies on a combination of clinical assessment and imaging studies. In rare or ambiguous cases, such as when a bursal lesion is unusually large, hemorrhagic, or atypically located, histopathological examination may be necessary to rule out neoplasm or infection. MRI is especially valuable for assessing the extent of the lesion and distinguishing between cystic (bursal) and solid masses, as well as identifying associated features like bone marrow edema or adjacent soft tissue inflammation. Most common types of bursitis are prepatellar, olecranon, trochanteric, and retrocalcaneal, with prepatellar and olecranon bursitis being especially frequent due to their superficial location and susceptibility to trauma or repetitive pressure [64–66] (Fig. 7; Table 3). Indeed, superficial bursitis, particularly at the elbow and knee, often results from chronic microtrauma (such as kneeling or leaning on elbows), but can also be caused by acute injury, inflammatory conditions like gout or rheumatoid arthritis, or infection (septic bursitis).

**Table 3:** Ultrasound assessment and management of different soft-tissue lesions

Soft-tissue Lesion	Location	How to Scan	Ultrasound Appearance	Doppler Findings	Clinical Notes	When to Escalate
Bursitis	Most common: prepatellar, olecranon, anserine, trochanteric, retrocalcaneal	Assess in two orthogonal planes for compressibility and wall features	Anechoic or hypoechoic, compressible fluid collection within a bursa. Chronic cases may show wall thickening, septations, or internal debris.	May show wall hyperemia	Differentiation from joint effusion or cellulitis is critical; US guides aspiration or injection.	MRI if contents atypical or recurrent; consider aspiration/injection or orthopedics consult if symptomatic or septic concern.
Septic arthritis	Joint space (commonly knee, hip, shoulder, elbow)	Check all joint recesses; compare with contralateral side; use color Doppler to assess synovial vascularity	Joint effusion, synovial thickening, periarticular soft tissue changes; US can detect early effusions before X-ray changes.	Increased synovial vascularity in inflamed joints	Medical emergency; early diagnosis and US-guided arthrocentesis essential to prevent joint damage	Immediate orthopedic/rheumatology consult for arthrocentesis and antibiotics; Escalation to CE MRI/CT is recommended when US findings are inconclusive, there is suspicion of deeper or adjacent bone involvement (such as osteomyelitis), or when the clinical picture is severe or not improving. Immediate antibiotic empiric therapy when there is a strong clinical suspicion of sepsis
Soft tissue infection	Subcutaneous tissue, fascial planes, deeper compartments	Check carefully the fascia; gentle probe compression helps distinguish abscesses from cellulitis; use color Doppler to assess for increased vascularity	*Cellulitis*: cobblestone pattern with hypoechoic reticulation; *Abscess*: well-defined fluid collection with debris and posterior enhancement; *Necrotizing fasciitis*: fascial fluid, subcutaneous gas (echogenic foci with shadowing).	*Cellulitis*: diffuse signs of hyperemia *Abscess*: hypervascular rim, avascular core *Necrotizing fasciitis*: variable	US improves diagnostic accuracy over clinical exam; anatomical detail (e.g., suprafascial vs. intramuscular) informs treatment.	Escalation to imaging (CE MRI or CT) or urgent orthopedic/surgical consult is warranted when there is high clinical suspicion for necrotizing infection. Imaging should not delay surgical intervention if clinical suspicion is high, as early and aggressive surgical debridement is critical for survival
Hematoma	Any soft tissue; common in trauma, anticoagulated patients	Evaluate in multiple planes; assess the size, shape, and extent of the hematoma, together with its compressibility and vascularity on Color Doppler	Variable: acute hematomas are hyperechoic; subacute become anechoic or show fluid-fluid levels; chronic may appear complex, septated, or calcified.	Typically avascular; vascular flow may be detected in cases of active bleeding.	May mimic tumors; compressibility and absence of vascularity help distinguish; follow-up or MRI recommended when diagnosis is uncertain.	CECT if persistent, enlarging, or masslike; urgent surgical/interventional if active bleeding or compartment syndrome suspected.
Morel-Lavallée lesion	Between subcutaneous tissue and fascia over the greater trochanter, thigh, knee, lumbar region, scapula	Scan along fascial interface; assess the size, shape, and extent of the lesion together with its compressibility and vascularity on Color Doppler	Fluid compressible collection between subcutaneous fat and fascia. Acute: echogenic, irregular; Chronic: hypoechoic or anechoic, well-defined, often flat or fusiform; may be encapsulated.	Avascular	Often missed in trauma; can progress to chronic pseudocyst or infection; treatment guided by stage and imaging features.	MRI if chronic or atypical; surgical consult if rapid expansion, infection, pseudocyst or skin necrosis risk.

#### Septic arthritis

Septic arthritis is a medical emergency characterized by infection within a joint. Staphylococcus aureus is the most common causative organism, but other bacteria, viruses, and fungi can also be responsible [67].The condition can rapidly evolve, leading to cartilage destruction and eventually to permanent joint damage or systemic complications if not diagnosed and treated promptly. Therefore, early recognition, accurate diagnosis, and aggressive management are essential to improve outcomes and reduce the risk of long-term disability or death. Septic arthritis typically arises from hematogenous spread, direct inoculation, or extension from nearby infections. Major risk factors include advanced age, diabetes, rheumatoid arthritis, immunosuppression, recent joint surgery, prosthetic joints, and intravenous drug use [67]. Patients usually present with acute monoarticular joint pain, swelling, redness, and limited movement [68]. Fever may be absent. Diagnosis relies on clinical suspicion, supported by elevated inflammatory markers (ESR, CRP), but definitive diagnosis requires synovial fluid analysis and culture [67]. US reliably detects joint effusions, synovial thickening, and soft tissue changes associated with septic arthritis, often before changes appear on X-ray [69]. It is highly sensitive for identifying effusions, even in subtle or early cases, and can distinguish between superficial infections and deeper joint involvement. An appropriate patient positioning is essential to maximize joint exposure while ensuring adequate comfort during US examination. For hip examinations, the supine position with external rotation is generally recommended, whereas a slight degree of flexion is preferable for the evaluation of the knee and elbow. Systematic assessment should be performed in both longitudinal and transverse planes to provide a comprehensive evaluation of the joint capsule and recesses and periarticular soft tissues. The recognition of specific anatomical landmarks, such as the femoral neck in the hip or the olecranon in the elbow, is helpful to orient the examination and to ensure reproducibility. US has also a role in guiding the arthrocentesis, allowing real-time visualization of the needle trajectory, thereby increasing the accuracy of intra-articular access and reducing the risk of complications [70]. However, US guided arthrocentesis should not delay antibiotic empiric therapy when there is a strong clinical suspicion of sepsis, as urgent treatment is critical to prevent morbidity and mortality [71].

#### Soft tissue infections

Sonographic evaluation of soft tissue infections helps distinguish between conditions like cellulitis, necrotizing fasciitis, and abscess, which can appear similar on physical exam but require different treatments. In cellulitis, US typically reveals a “cobblestoning” pattern with hypoechoic, reticulated areas in the subcutaneous tissue reflecting interstitial fluid, but without a discrete fluid collection; Doppler imaging demonstrates signs of hyperemia, helping differentiation from simple soft-tissue edema. Abscesses appear as well-defined, hypoechoic or anechoic fluid collections, often with posterior acoustic enhancement and sometimes with internal debris that may move with compression [72]. The walls of an abscess may appear irregular and thickened. As the lesion matures, Doppler imaging often demonstrates a hypervascular capsule surrounding an avascular core. It is essential to report the precise location of the abscess, particularly distinguishing whether it is suprafascial or intramuscular, as this may have implications for treatment planning. US has demonstrated high sensitivity and specificity for detecting abscesses compared to clinical examination alone, especially when the diagnosis is uncertain [73, 74]. Necrotizing fasciitis present as subcutaneous thickening with fluid tracking along fascial planes and subcutaneous gas, seen as bright echoes with shadowing: the presence of gas is a key feature that help differentiate necrotizing fascitis from cellulitis and soft tissue edema, enabling early diagnosis and intervention [75]. Overall, the sensitivity of US for the diagnosis of necrotizing fasciitis ranged from 85% to 100%, while specificity ranged from 44% to 98% [76].

#### Soft tissue hematoma

A soft tissue hematoma is a localized collection of blood within the soft tissues, typically resulting from trauma, surgical procedures, or as a complication of anticoagulant therapy. Its clinical significance varies widely, ranging from minor lesions to potentially life-threatening conditions, depending on the size and anatomical location. In recent years, spontaneous soft tissue hematomas have become increasingly common, largely due to the growing use of anticoagulants. Computed tomography angiography (CTA) is the primary imaging modality for evaluating the extent of hemorrhage and identifying active bleeding. It plays a crucial role in guiding treatment decisions, including arterial embolization, which is associated with high rates of both technical and clinical success [77]. Conventional US is preferred over CT for evaluating soft tissue hematomas when the lesion is superficial, easily accessible, and the clinical question is focused on initial diagnosis or follow-up, especially in settings where minimizing radiation exposure is important, such as in children, pregnant patients, or for serial monitoring. Conventional US is effective for hematoma detection but limited in identifying active bleeding, whereas contrast-enhanced US significantly improves diagnostic accuracy by enabling real-time visualization of microbubble extravasation as a direct marker of ongoing hemorrhage [78–81].On US, soft tissue hematomas typically present as well-defined, anechoic or heterogeneous masses. Their sonographic appearance evolves over time as the blood clots and undergoes organization. In the hyperacute phase, hematomas often appear hyperechoic due to fresh coagulated blood. As the clot begins to liquefy, the lesion may become anechoic and increase in size. With further evolution, hematomas often become more complex, exhibiting internal septations and fluid-fluid levels, reflecting ongoing clot lysis and organization. In the chronic phase, hematomas may display a more cystic morphology, with well-defined margins and occasional calcifications, or may present a solid structure due to the presence of the different products of hemoglobin (Fig. 7). On US, the compressibility of a hematoma depends on its stage and composition. In the acute phase, hematomas may be somewhat compressible, but as they evolve and organize, they become less compressible. This distinction is important for diagnosis and management, as compressibility can help differentiate a hematoma from other soft tissue masses or abscesses. Differentiating hematomas from soft tissue tumors, such as sarcomas, can be challenging, as both may appear as mass-like lesions with overlapping sonographic features. Hematomas typically lack internal vascularity on Doppler imaging, whereas tumors often demonstrate internal blood flow. In the emergency setting, follow-up imaging is recommended, and MRI should be considered when the diagnosis is uncertain. Misdiagnosis is not uncommon; reports have shown that soft tissue sarcomas are sometimes mistaken for hematomas, leading to diagnostic delays and poorer outcomes [82–84].

#### Morel-Lavallee lesion

A Morel-Lavallée lesion is a closed soft-tissue degloving injury caused by severe trauma that shears the skin and subcutaneous tissue away from the underlying fascia, creating a potential space that fills with blood, lymph, and debris [85]. These lesions most commonly occur over the greater trochanter of the femur and in the thigh, but can also be found in other areas such as the knee, lumbar spine, and scapula [85]. Early recognition is critical, as Morel-Lavallée lesions are often overlooked or misdiagnosed, particularly in polytrauma patients, and may progress to infection, skin necrosis, or chronic pseudocyst formation if not properly treated [85–87]. On US, Morel Lavallee lesion typically appears as a compressible fluid collection located between the deep fat and fascia, often with well-defined margins as the lesion ages [88]. Acutely, they are often echogenic due to clotted blood, becoming hypoechoic or anechoic as contents liquefy [89] (Fig. 7). A capsule of variable thickness may be present. Acute lesions tend to be heterogeneous, lobular, and irregular, while chronic ones are more homogeneous, well-circumscribed, and typically flat or fusiform [90]. There are currently no universally accepted treatment guidelines, but algorithms have been proposed to guide management based on lesion characteristics [86–91].

#### Occulte fractures

**Fig. 8:**
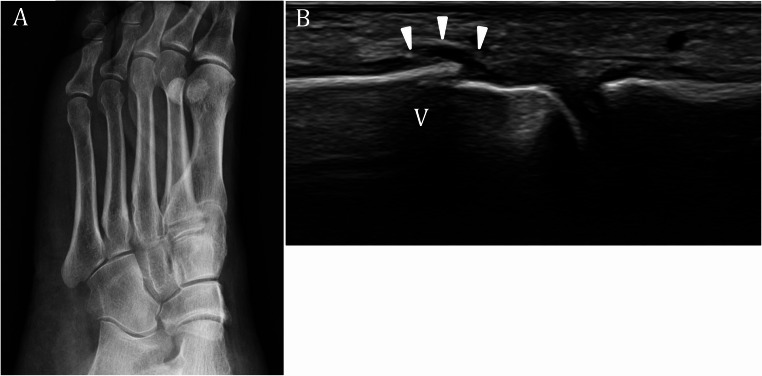
Occult fracture of the fifth metatarsal bone. **A**. Negative radiographic examination of the foot performed after a trauma. **B**. On long axis US image, an interruption (arrowheads) of the cortical profile of the fifth metatarsal bone (V) is visible, consistent with a fracture

Occult fractures are those not visible on initial radiographs but later confirmed by other imaging modalities or follow-up studies [92, 93]. Multiple systematic reviews and cohort studies have demonstrated high sensitivity and specificity of US in detecting radiographically occult fractures, particularly of the scaphoid, ankle, foot, and in pediatric populations [94–98]. In children, US can depict cortical discontinuity and indirect signs of fracture, such as joint effusion, with direct visualization reported in over 90% of cases, making it a valuable radiation-free alternative, especially for long bones (Fig. 8). In the knee, lipohemarthrosis detected by US has been proven to be a highly sensitive and specific indirect sign of intra-articular fracture, with a study showing greater sensitivity and specificity compared to radiographs, enabling early detection of occult fractures not visible on initial radiographs [99].However, US is operator-dependent and may be less reliable for small or complex bones. Therefore comparative imaging or follow-up with CT/MRI is recommended if US findings are inconclusive or if clinical suspicion remains high.

## Conclusion

This review focused on high-yield applications of MSK US that are most relevant to general radiologists working in emergency settings, including the assessment of tendon ruptures, muscle injuries, bursitis, soft tissue infections, and hematomas. Nerve injuries were not addressed, as their evaluation typically requires more advanced skills and detailed anatomical knowledge, making them less feasible in routine emergency radiology practice. Although not all of the conditions described in the article are frequently encountered, they represent clinically significant scenarios in which US can play a crucial role. With appropriate training, a basic US examination can be effectively performed in most of these cases, supporting timely diagnosis and facilitating appropriate management.

## Data Availability

Not applicable. No new data were generated or analyzed for the purposes of this review.
